# Treatment of traumatized refugees with Sertraline versus Venlafaxine in combination with psychotherapy – study protocol for a randomized clinical trial

**DOI:** 10.1186/1745-6215-14-137

**Published:** 2013-05-11

**Authors:** Charlotte Sonne, Jessica Carlsson, Ask Elklit, Erik Lykke Mortensen, Morten Ekstrøm

**Affiliations:** 1 Psychiatric Trauma Clinic for Refugees, Gentofte Hospital, opg. 32, st. Niels Andersens vej 65, 2900 Hellerup, Denmark; 2 National Center for Psychotraumatology, Campusvej 55, 5230 Odense, Denmark; 3 Institute of Public Health and Center for Healthy Aging, University of Copenhagen, Øster Farimagsgade 5, 1353 København K, Denmark

**Keywords:** Refugee, PTSD, Depression, Trauma, Venlafaxine, Sertraline

## Abstract

**Background:**

Sufficient evidence is lacking to draw final conclusions on the efficiency of medical and psychological treatments of traumatized refugees with PTSD. The pharmacological treatments of choice today for post-traumatic stress disorder are antidepressants from the subgroup selective serotonin reuptake inhibitors, especially Sertraline. The evidence for the use of selective serotonin reuptake inhibitors in the treatment of complex post-traumatic stress disorder in traumatized refugees is very limited. Venlafaxine is a dual-action antidepressant that works on several pathways in the brain. It influences areas in the brain which are responsible for the enhanced anxiety and hyper-arousal experienced by traumatized refugees and which some studies have found to be enlarged among patients suffering from post-traumatic stress disorder.

**Design:**

This study will include approximately 150 patients, randomized into two different groups treated with either Sertraline or Venlafaxine. Patients in both groups will receive the same manual-based cognitive behavioral therapy, which has been especially adapted to this group of patients. The treatment period will be 6 to 7 months. The trial endpoints will be post-traumatic stress disorder and depressive symptoms and social functioning, all measured on validated ratings scales. Furthermore the study will examine the relation between a psycho-social resources and treatment outcome based on 15 different possible outcome predictors.

**Discussion:**

This study is expected to bring forward new knowledge on treatment and clinical evaluation of traumatized refugees and the results are expected to be used in reference programs and clinical guidelines.

**Trial registration:**

ClinicalTrials.gov NCT01569685

## Background

The treatment of traumatized refugees is one of the least researched areas within the field of psychiatry. Most research on post-traumatic stress disorder (PTSD) and other psychiatric conditions related to traumatic stress have been carried out on victims of other kinds of traumatic experience such as rape, traffic accidents or trauma related to war. There are reasons to believe that mental symptoms experienced after trauma relates to the type and intensity of the trauma. Most traumatic events (traffic accidents, robberies, natural disasters, et cetera) are of limited duration. However, many refugees experience more or less constant or repeated trauma for months or even years, such as periods with daily exposure to torture during a long imprisonment. The current PTSD diagnosis often does not fully capture the severe psychological harm that occurs with such prolonged trauma. People such as refugees experiencing long-lasting trauma often report additional symptoms alongside formal PTSD symptoms, such as alterations in emotional control and dissociative symptoms. Although not a formal diagnosis in either the Diagnostic and Statistical Manual of Mental Disorders (DSM) or the International Classification of Diseases (ICD) diagnostic system, this is often referred to as complex PTSD by clinicians and researchers within the field.

Apart from a history of repeated trauma in their country of origin, the process of forced immigration experienced by refugees is often traumatic and dramatically impacts the lives of individuals and families [1,2]. It also tears apart the social structures that are often very important to people from highly collectively minded societies. Furthermore, the ways of understanding mental health problems often differ from the country of origin to the receiving country. With this in mind, the research results from studies conducted on other patient populations cannot simply be transferred to traumatized refugees. It is therefore problematic that the three available Cochrane analyses of PTSD have only been able to identify very few studies of traumatized refugees [3-5].

The Danish Medical Technology Report (MTV report), *The treatment and rehabilitation of PTSD inclusive traumatized refugees*[6], concluded that antidepressants from the subgroup of selective serotonin reuptake inhibitors (SSRIs), among these the drug Sertraline, are currently the drugs of choice for the treatment of PTSD. However the report also concluded that SSRIs are inadequate as a treatment for complex PTSD. Some antidepressants from the subgroup of dual-action products, among these Venlafaxine, have shown promising results in clinical case reports [7] but have not been investigated thoroughly in randomized studies on traumatized refugees. Studies of other groups of PTSD patients conclude that both short-term and long-term Venlafaxine treatment is effective in PTSD [8,9]. Although the exact pathophysiology of PTSD still remains to be fully understood, the brain’s noradrenergic pathways are recognized to be involved. Venlafaxine acts on both serotonin and norepinephrine pathways in many areas in the brain. Among other structures it is believed to play a role in the regulation of the amygdala, which is currently held at least partly responsible for the enhanced anxiety and hyper arousal experienced by traumatized refugees. This gives us reason to believe that this drug could provide greater relief of cluster D PTSD symptoms in particular.

A Cochrane review from 2010 of combined pharmacological and psychological treatment of PTSD included only four studies, all conducted on a very limited number of patients [4]. The authors concluded ‘Further research into the clinical management of PTSD is required including large trials that use (i) reliable and clinically meaningful outcome measurements such as remission of PTSD, (ii) consistent measures of PTSD symptoms and (iii) functional outcomes, including those related to social and occupational function’. Furthermore, the authors called for studies of homogeneous populations such as traumatized refugees.

The latest Cochrane review, as well as the MTV report mentioned above, consider social functioning to be an important issue, which could potentially be a trial endpoint on the same level as symptoms ratings [4,6]. Related to this issue is the current lack of understanding of the relationship between psychosocial resources and treatment outcome.

### Objectives

The objectives are to: 1) examine differences in the treatment outcome of patients treated with Venlafaxine and Sertraline respectively; 2) study the relationship between changes in symptoms in PTSD /depression and changes in social functioning from baseline to post treatment evaluation; 3) investigate if pre-treatment ratings of patient resources correlate with the actual outcome of the treatment for the individual patient.

## Methods

The study is a randomized clinical trial aiming to include approximately 200 traumatized refugees of which a minimum of 150 are estimated to complete the trial in accordance with the protocol. Patients are randomized into two groups treated with either Sertraline or Venlafaxine in combination with manual-based cognitive therapy, as described below. The treatment period is 6 to 7 months. Treatment outcome is evaluated in two ways, through self-ratings and blinded observer-ratings. Patients complete self-ratings on three occasions during the study; these are at the pre-treatment consultation (baseline), between phase 1 and 2 (please see below), and at the end of the treatment period. Blinded observer-ratings are carried out by a team of trained medical students who do not know to which intervention group the patient has been randomized. These ratings are carried out at the beginning and at the end of the treatment period. The rating scales are described in detail in the outcome section below.

### Participants

The study is being carried out at the Psychiatric Trauma Clinic for Refugees (PTF), which is part of the Psychiatric Center Ballerup, situated in the Capital Region of Denmark. The different effects of two medical treatments in combination with psychotherapy are being studied on a relatively homogenous group of adult (≥ 18 years of age) traumatized refugees who are referred to the PTF during the study period. Social functioning will be included as one of the trial endpoints and psychosocial predictors and their relation to treatment outcome will be analyzed.

The study will ultimately include approximately 200 patients over a period of 15 months. About 150 patients are expected to complete the study (based on previous studies at the PTF in a similar group of patients). Patients are either referred to the PTF by their general practitioner (GP) or by a referring doctor in a specialist unit. If it seems likely that the patient belongs to the target group of the clinic, he or she will be invited to a pre-treatment consultation.

#### Inclusion criteria

The inclusion criteria are as follows: patients must be referred to PTF between 1 April 2012 and 31 June 2013; ≥ 18 years of age; have symptoms of PTSD in accordance with the ICD-10 research criteria; have had psychological trauma in the past; be motivated to undergo treatment; provide written informed consent.

#### Exclusion criteria

Patients will be excluded if they are suffering from serious psychotic disorder (defined by ICD-10 diagnosis F2x and F30.1-F30.9); are currently abusing drugs or alcohol (ICD-10 F.1×.24-F1×.26); are in need of admission to a psychiatric hospital; do not give written informed consent, or are pregnant, breast feeding or plan to become pregnant during the project period.

If the patient fulfils the inclusion criteria, and is not excluded by the exclusion criteria, the patient is included and randomized to one of two intervention groups:

### Treatment groups

**Figure 1:**
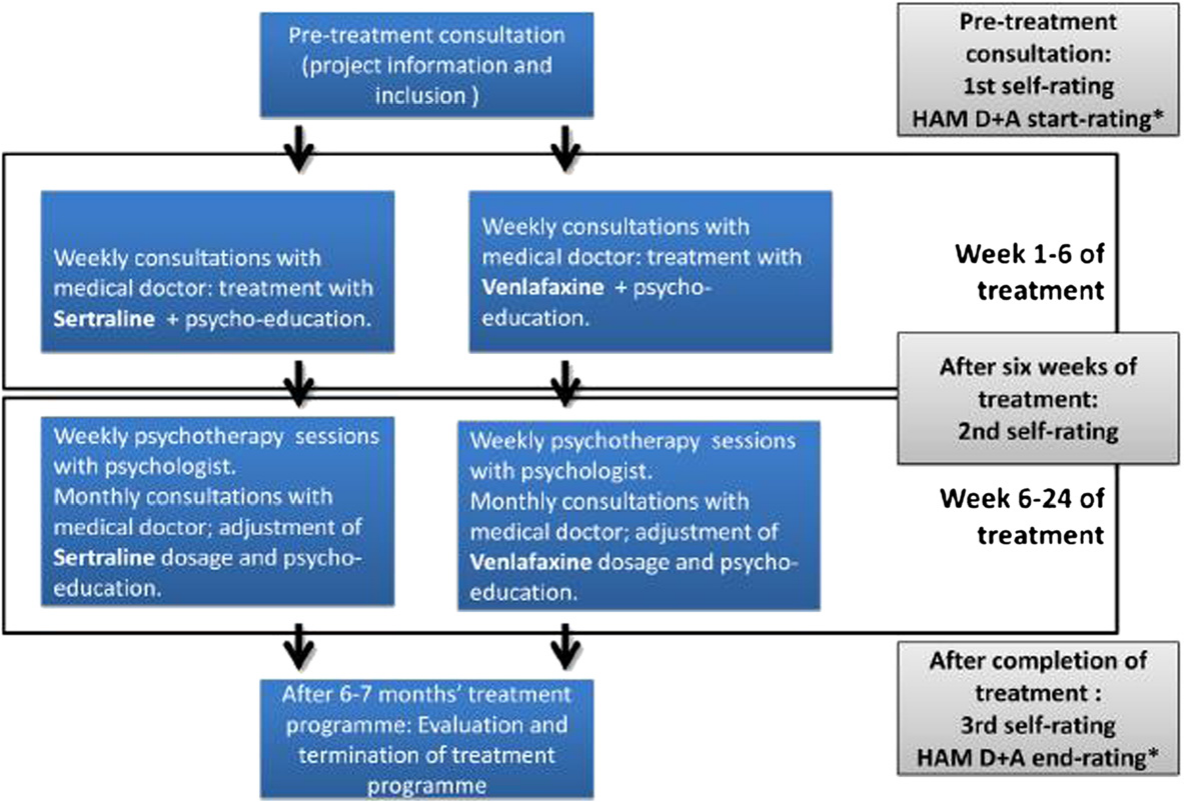
Treatment and data collection.

Group V1 is assigned to treatment with Venlafaxine and manualised psychotherapy developed to fit the target group of the clinic. Group S2 is assigned to treatment with Sertraline and manualised psychotherapy developed to fit the target group of the clinic. In both groups treatment is planned to last between 6 and 7 months. The treatment and the ratings are shown in Figure 1. The different parts of the intervention are described in detail below.

### The intervention

The intervention consists of a combination of medical, psychological and psychosocial treatment and is divided into two phases. During phase 1 (the first 2 months) the patient only has consultations with the doctor and receives no psychological treatment. During phase 2 (the last 4 months) the patient has sessions with both the doctor and the psychologist as described below. The patient meets with the social counsellor at least twice during the study period. If extra consultations are agreed, these are registered in the patient record (for example, if there are medical or psychosocial problems that need immediate attention).

#### Medical intervention

Each patient is offered 10 sessions with one of the medical doctors at the PTF. Sessions are scheduled to take place weekly during phase 1 and monthly during phase 2. During these sessions the medical treatment of the randomized group is initiated and monitored. If the patient suffers from extensive sleeping problems, Mianserine (10 to 30 mg) is given additionally in both groups. If at all possible, patients are taken off all other kinds of psycho-pharmacological treatment. In small doses Mianserine is believed to act primarily on sleep disturbances, not on symptoms of depression. Furthermore, Mianserine is given for the same indication in both groups of patients. Mianserine should not affect our ability to analyze differences in treatment response for Sertraline and Venlafaxine respectively, as there is no pharmacological reason to believe that Mianserine interacts differently with either of the two drugs being compared in this trial.

In addition to the medical treatment, the patients receive psycho-education on topics such as symptoms of PTSD and depression; the rationale for treatment; healthy lifestyle, including exercise and proper diet; breathing and relaxation exercises; sleep disturbances, and chronic pain. Twelve different one-page handouts are available at PTF on a range of these topics (in five different languages). They are given to the patients to take home when a topic has been discussed during a treatment session. The patient and the doctor together decide the relevant topic for each session.

The medical and psycho-educational treatment is thoroughly described in a treatment manual followed by all doctors. The main topic of each session is registered in the patient record together with side effects of the treatment and any social problems that the patient has. Furthermore, a brief psychiatric evaluation is carried out by the doctor and noted using a tick-box system developed at PTF and used in previous studies.

#### Psychological intervention

Each patient is offered one introductory session and sixteen therapeutic sessions with a psychologist. The introduction session is in phase 1, before or right at the beginning of the treatment period. The remaining sessions take place during phase 2.

All therapy is based on the same manual, which is primarily based on cognitive therapy with elements of trauma-focused therapy, acceptance commitment therapy (ACT) and mindfulness. The methods have been adapted to this patient group on the basis of the available literature and on experience of the three manuals previously used at the clinic.

After each session the therapist fills in a methodology scheme in the patient record, thereby registering the methods that were used during the session and whether the patient had completed planned homework. All psychologists regularly attend manual supervision sessions to ensure that therapy is in accordance with the manual and to avoid too much inter-therapist variation.

#### Psycho-social intervention

All patients are offered at least two appointments with a social counsellor during the project period: one at the beginning and one towards the end of the treatment. During these sessions patients are asked to complete two self-ratings that are related to social functioning and network; these are further described below. After the first session the social counsellor writes a letter to the patient’s contact at the social services office describing the social problems that the patient might have. All contacts to external parties, including the patient’s relatives, are registered in the patient record.

#### Group lectures

Group lectures are offered to all patients once a month. Three different lectures are offered, one on each of the following topics: the structure of the Danish municipal administration and social services; advice on financial debt and the tax system, and citizenship and possibilities for support and social activities. As lectures are offered every month in a fixed rotation the patients have two possibilities to attend each of the different lectures during a 6-month period.

### Outcome

Data collection combines self-ratings and observer-ratings. Patients are asked to fill in self-ratings three times: at their first appointment at PTF (baseline), immediately before they start on phase 2, and at the end of the treatment period. Patients and doctors are not treatment-blinded, but a team of specially trained intervention-blinded medical students are carrying out Hamilton anxiety and depression ratings at baseline and at completion of the treatment. Interpreters assist the patients during both ratings and treatment sessions when needed. All the rating scales used in the study are validated and have previously been used in different cultural settings. At PTF they are available in five different languages.

#### Primary outcome measure

The Harvard trauma questionnaire (HTQ) [10] is a self-administered rating scale used to monitor the severity of PTSD in different patient groups, among these, groups of traumatized refugees. It is internationally recognized and validated in several different languages. The first 16 questions in the HTQ part IV (symptoms) have been chosen to monitor PTSD symptoms, as they cover all PTSD symptoms in both DSM-IV and ICD-10.

#### Secondary outcome measures

The Hopkins symptom check list-25 (HSCL-25) [11] is a self-administered rating scale used to monitor the severity of anxiety and depression symptoms. It consists of 25 questions, 10 about anxiety symptoms and 15 about depression symptoms. The social adjustment scale self-report (SAS-SR) short version [12] is a self-administered rating scale used to monitor social functioning during treatment. It is a shorter version of a 54-question rating scale. The short form used in this study consists of 24 questions. The Hamilton depression and anxiety ratings scales (HAM D+A) [13] are observer-administered rating scales based on a semi-structured interview. They have been used for many years in different areas of psychiatry to monitor progression in depression and anxiety symptoms.

#### Other psychometric instruments used in the study

The following instruments areused: the WHO-5, a five-item self-administered rating scale used to monitor life quality in different groups of psychiatric patients; the somatisation items of Symptom Check List-90 (SCL-90), a self-administered rating scale broadly used in the psychiatric field, using the part of the scale that monitors somatic complaints; pain in four different body areas measured using a visual analogue scale (VAS), a self-administered rating scale used to monitor pain in four different body areas, namely the head, arms, legs and neck/back; the Sheehan disability scale (SDS), a self-administered rating scale comprising three different VAS scales used to measure three different areas of functioning, namely family life, work and social networks; global assessment of functioning (GAF), an observer-administered rating scale used to evaluate symptoms and functioning level in adults. In clinical settings the GAF is used to monitor treatment effect in many different groups of psychiatric patients; goal attainment scaling (GAS), which is used to make the individual patient state his/her own measures for successful treatment outcome in a way that makes it possible to use it for treatment evaluation, and finally, the crisis support scale (CSS), a seven-item self-rating scale used to monitor the social support experienced after a traumatic event in different groups of PTSD patients.

### Psycho-social resources and relation to treatment outcome

**Figure 2:**
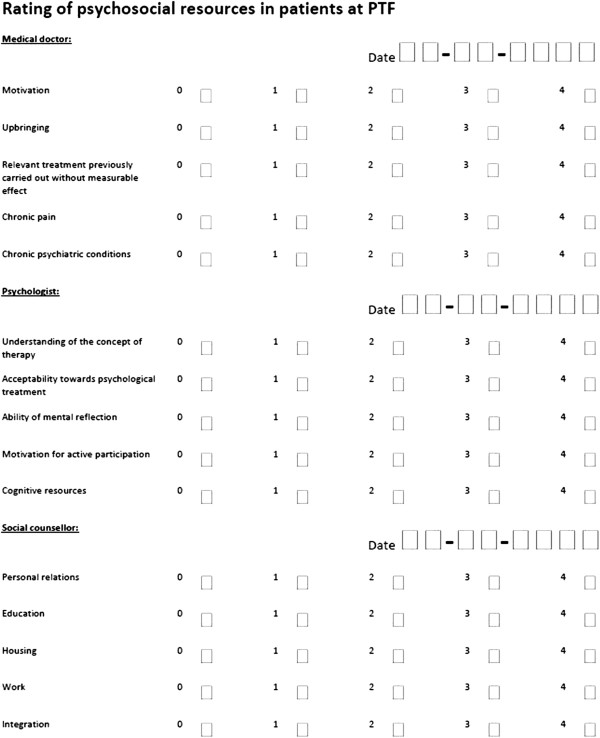
Rating of psychosocial resources in patients at the Psychiatric Trauma Clinic for Refugees.

If a patient agrees to participate in the treatment program a ratings table (Figure 2) is completed in accordance with a manual explaining the rating of each item, to secure inter-rater reliability. This is done during the first session with the patient, which is scheduled before the actual treatment or at the beginning of the treatment program. The doctor completes the first five questions, the psychologist completes the next five, and the last five questions are completed by the social counsellor. Based on the score, the patients are divided into three groups: high, moderate or limited psycho-social resources. These groups are not to be confused with the treatment groups.

The purpose of this part of the study is to investigate associations between psychosocial resources and treatment outcome. Consequently, after completed treatment, the patients are again divided into three groups, but this time in accordance with outcome (high, moderate or limited) measured by the primary outcome measure of the study. The psychosocial resource categorization is then compared with the outcome categorization in order to analyze whether there is a relation between psychosocial resources and the ability to benefit from the treatment. Furthermore, the individual rating items are analyzed to identify the strongest outcome predictors.

### Randomization

The randomization list is made by the Biostatistics Department at the University of Copenhagen, while the randomization of the individual patient is carried out by a group of secretaries in a division of the Psychiatric Center in Ballerup that is independent of the PTF. Before randomization, stratification is made by gender and baseline HTQ score.

### Data analysis

#### Sample size and power calculations

If the included 150 patients are divided into two groups of equal size, the power to detect a group difference of half of 1 SD on the rating scales will be 86%, while the power to detect a difference of 1 SD will be close to 100%. Differences of less than half of 1 SD are considered to be of marginal interest from a clinical point of view.

#### Statistical analysis

For analysis of objective 1, the outcome variables are the baseline, second and third ratings in the primary and secondary outcome measures. With two intervention groups and three ratings, the design of the study may be described as a 2 × 3 factorial design with repeated measures of the last factor. Analysis of variance (ANOVA) with repeated measures of one factor can be used to analyze this design, but multi-level analysis will be more efficient if there are missing observations on one or more of the three ratings. In these analyses the main effect of the rating factor represent changes during the course of therapy, but the main focus is the interaction between the group and rating factor, corresponding to group differences in patterns of mean ratings. If difference scores are calculated between the three ratings, group differences in differences scores correspond to this interaction. Group differences in multiple difference variables can be analyzed by multivariate analysis of variance (MANOVA), while single difference variables (for example, between the third rating and the baseline score) can be tested with a *t*-test for independent groups. Adjustment for the effect of baseline values on outcome variables and background factors (such as gender and age) can be done using analysis of covariance (ANCOVA)/linear regression, including multi-level models. Intention-to-treat analyses will be carried out alongside completer analyses.

For analysis of objective 2, the relationship between changes in social functioning and changes in symptoms can be calculated by bivariate or partial correlations, if adjustments are made for baseline or background factors as described above. For objective 3, from the baseline predictor table patients are divided into three groups according to the predicted treatment outcome and using relevant statistical methods. This categorization is compared with a categorization based on the actual treatment outcome. The degree of concordance can be evaluated with kappa coefficients or correlation coefficients based on the uncategorized ordinal measures of predicted and actual treatment outcome.

### Ethics

The trial protocol has been approved by The Ethics Committee of the Capital Region of Denmark, the Danish Medicines Agency and the Danish Data Protection Agency. The project recognizes the Helsinki II Declaration. Participation in the project is voluntary and requires written, informed consent. Patients who do not wish to participate in the project still have the right to receive equal treatment at the PTF. Randomization is considered ethical, as current evidence cannot clarify whether one of the treatments offered to the patients is better than the other.

### Publications

Three publications are planned after the conclusion of the data collection to describe 1) the effect of Sertraline versus Venlafaxine on trauma-related psychiatric disorder in refugees; 2) changes in social functioning in relation to PTSD and depression symptoms during a six-month treatment program for traumatized refugees, and 3) the relationship between psychosocial resources and treatment outcome in traumatized refugees.

### Trial status

Patient inclusion in this trial started on 1 April 2012 and is scheduled to continue until 31 June 2013.

## Abbreviations

ACT: acceptance and commitment therapy; ANCOVA: analysis of covariance; ANOVA: analysis of variance; CSS: crisis support scale; GAF: global assessment of functioning; GAS: goal attainment scaling; HAM-A: Hamilton anxiety scale; HAM-D: Hamilton depression scale; HSCL-25: Hopkins symptom check list; HTQ: Harvard trauma questionnaire; ICD-10: International classification of diseases-10; MANOVA: multivariate analysis of variance; MTV: Medical Technology Report; PTF: Psychiatric Trauma Clinic for Refugees; PTSD: post-traumatic stress disorder; SAS-SR: social adjustment scale - self report; SDS: Sheehan disability scale; SSRI: selective serotonine reuptake inhibitor; VAS: visual analogue scale.

## Competing interests

The authors CS, JC, ME, AE and ELM hereby declare that they have no competing interests.

## Authors’ contributions

CS is the primary investigator of the study, designed the study and is the primary author of the protocol as well as this manuscript. JC is a co-investigator in the study, participated in the design of the study and helped to draft the protocol and this manuscript. AE helped to draft the protocol and has revised the manuscript critically. ME and ELM have been involved in the design of the study and helped to draft the protocol and the manuscript. All authors read and approved the final manuscript.

## References

[B1] Laban CJ Gernaat HBPE Komproe IH Van der Tweel I De Jong JTVM Postmigration living problems and common psychiatric disorders in Iraqi asylum seekers in the Netherlands J Nerv Ment Dis 2005 193 825 832 10.1097/01.nmd.0000188977.44657.1d 16319706

[B2] Lindencrona F Ekblad S Hauff E Mental health of recently resettled refugees from the Middle East in Sweden: the impact of pre-resettlement trauma, resettlement stress and capacity to handle stress Soc Psychiatry Psychiatr Epidemiol 2008 43 121 131 10.1007/s00127-007-0280-2 18060523

[B3] Stein DJ Ipser J Seedat S Pharmacotherapy for post traumatic stress disorder ( PTSD ) ( Review ) Cochrane Database Syst Rev 2009 10.1002/14651858.CD002795.pub2 PMC6993948 16437445

[B4] Hetrick S Purcell R Garner B Parslow R Combined pharmacotherapy and psychological therapies for post traumatic stress disorder ( PTSD ) ( Review ) Cochrane Database Syst Rev 2010 10.1002/14651858.CD007316.pub2 PMC12515543 20614457

[B5] Bisson J Andrew M Psychological treatment of post-traumatic stress disorder (PTSD) ( Review) Cochrane Database Syst Rev 2009 10.1002/14651858.CD003388.pub3 17636720

[B6] Region Syddanmarks Center for Kvalitet MTV om behandling og rehabilitering af PTSD – herunder traumatiserede flygtninge 2008

[B7] Hamner MB Frueh BC Response to venlafaxine in a previously antidepressant treatment-resistant combat veteran with post-traumatic stress disorder Int Clin Psychopharmacol 1998 13 233 234 10.1097/00004850-199809000-00008 9817630

[B8] Davidson J Baldwin D Stein DJ Kuper E Treatment of posttraumatic stress disorder with venlafaxine extended release Arch Gen Psychiatry 2006 63 1158 1165 10.1001/archpsyc.63.10.1158 17015818

[B9] Davidson J Rothbaum BO Tucker P Asnis G Benattia I Musgnung JJ Venlafaxine extended release in posttraumatic stress disorder: a sertraline- and placebo-controlled study J Clin Psychopharmacol 2006 26 259 267 10.1097/01.jcp.0000222514.71390.c1 16702890

[B10] Hollifield M Measuring trauma and health status in refugees: a critical review JAMA 2002 288 611 621 10.1001/jama.288.5.611 12150673

[B11] Mollica RF Wyshak G De Marneffe D Khuon F Lavelle J Indochinese versions of the Hopkins Symptom Checklist-25: a screening instrument for the psychiatric care of refugees Am J Psychiatry 1987 144 497 500 3565621 10.1176/ajp.144.4.497

[B12] Weissman MM Olfson M Gameroff MJ Feder A Fuentes M A comparison of three scales for assessing social functioning in primary care Am J Psychiatry 2001 158 460 466 10.1176/appi.ajp.158.3.460 11229989

[B13] Hamilton M A rating scale for depression J Neurol Neurosurg Psychiatry 1960 23 56 62 10.1136/jnnp.23.1.56 14399272 PMC495331

